# Supplementary figures and images for: The Candida auris Hog1 MAP kinase is essential for the colonization of murine skin and intradermal persistence
Source: mBio. 2024 Oct 18;15(11):e02748-24. doi: 10.1128/mbio.02748-24 (PMC11558994; doi:10.1128/mbio.02748-24)

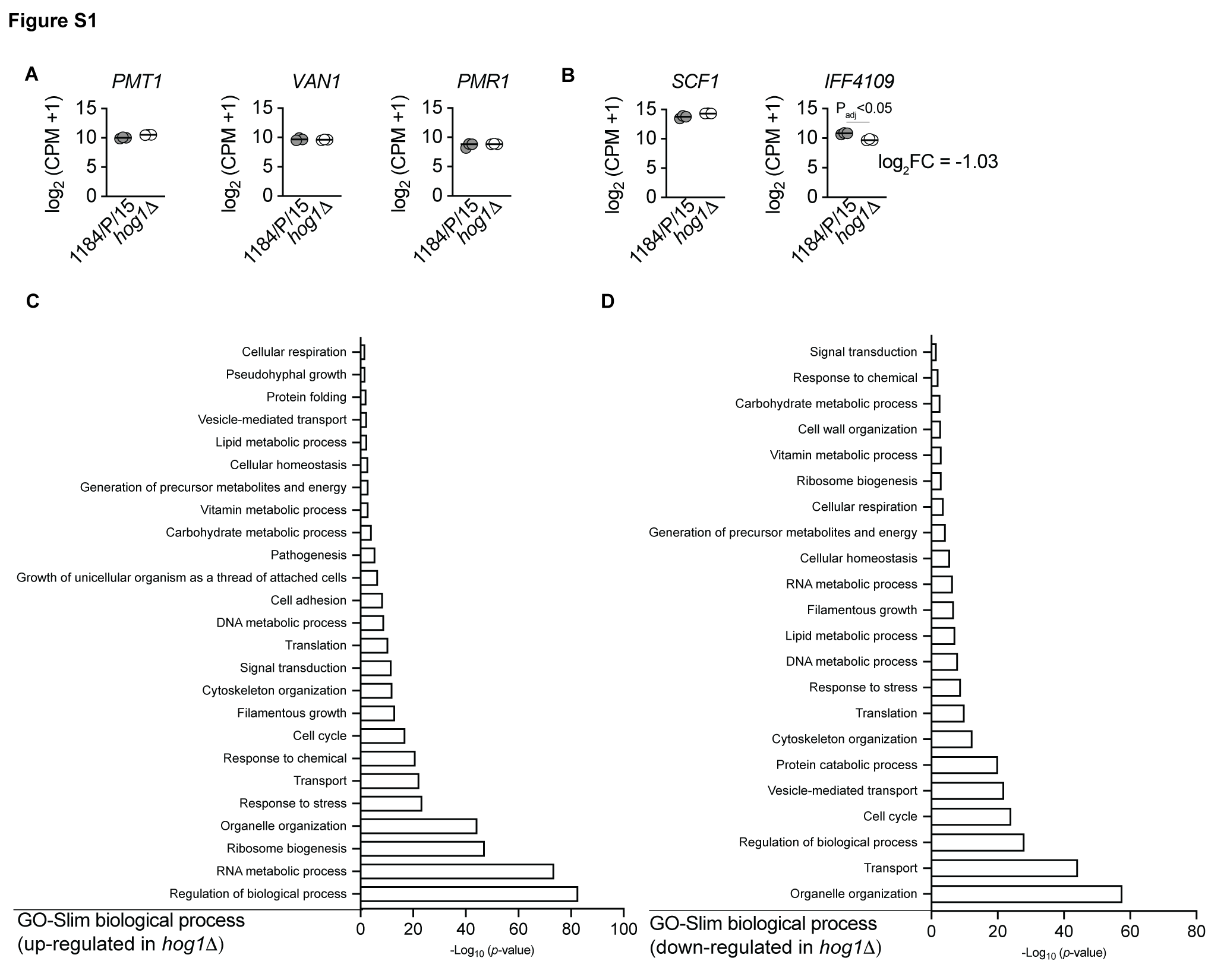

Supplement: Fig. S1 — Transcript levels of genes involved in mannosylation and adhesion. [file mbio.02748-24-s0001.tif]

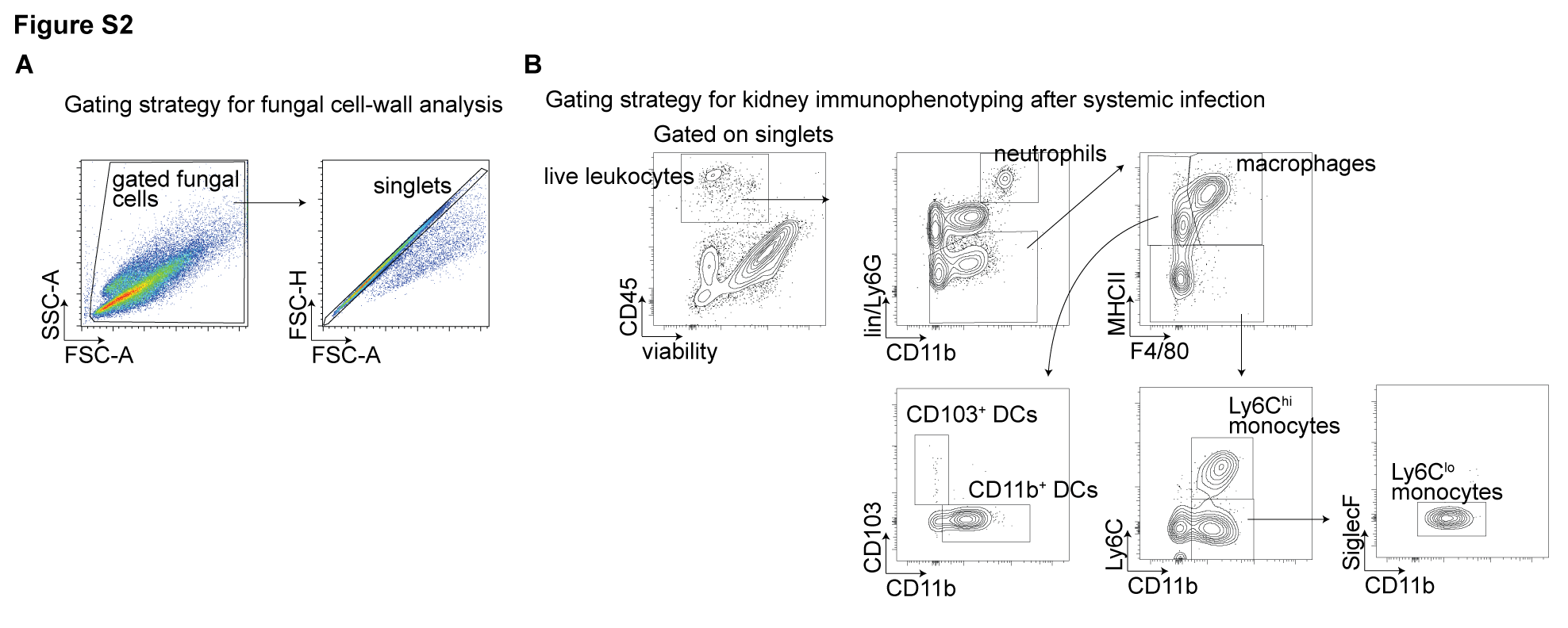

Supplement: Fig. S2 — Gating strategy and immunophenotyping of C. auris-infected kidneys. [file mbio.02748-24-s0002.tif]

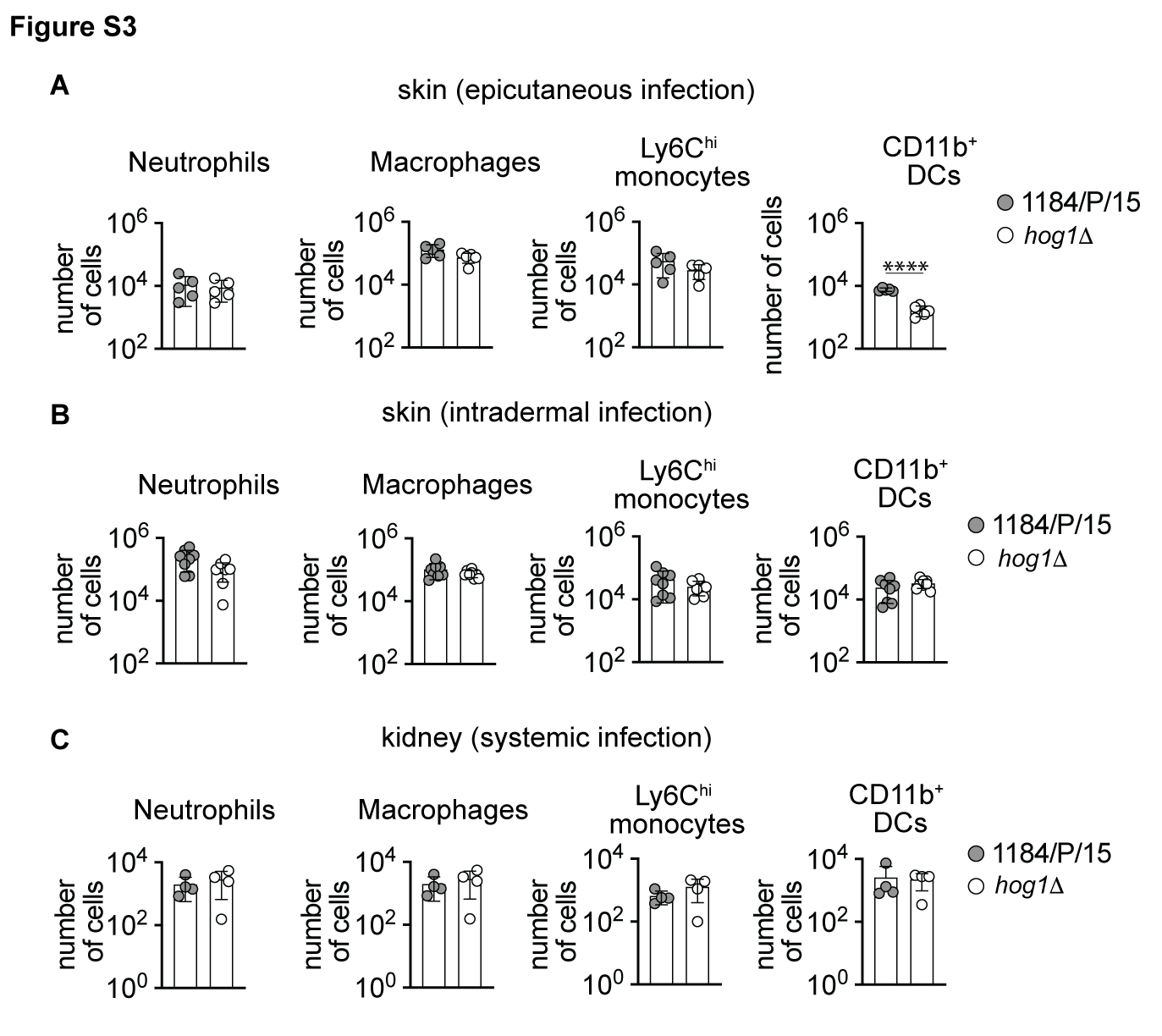

Supplement: Fig. S3 — Myeloid phagocyte accumulation of C. auris-infected murine skin and kidneys. [file mbio.02748-24-s0003.tif]
