# Supplementary material for: The Candida auris Hog1 MAP kinase is essential for the colonization of murine skin and intradermal persistence
Source: mBio. 2024 Oct 18;15(11):e02748-24. doi: 10.1128/mbio.02748-24 (PMC11558994; doi:10.1128/mbio.02748-24)
Supplement: Legends — Supplemental material legends. [file mbio.02748-24-s0004.docx]

**Supplemental Material Legends**

**Table S1.** List of differentially expressed genes in the *hog1*Δ mutant strain

**Supplementary Figure Legends**

**FIG S1: Transcript levels of genes involved in mannosylation and adhesion.**

**(A-B)** Gene expression profiles of *C. auris* isolates grown in YPD were determined using RNA-seq of three biological replicates. Log-transformed normalized counts (CPM+1) for indicated genes (*PMT1* *(B9J08_000681), VAN1(B9J08_003572), PMR1 (B9J08_000837), SCF1(B9J08_001458), IFF4109 (B9J08_004109)* depicts the variation of *HOG1* expression in parent and *hog1*Δ cells. Dots represent individual replicate and horizontal line indicates mean. Approximately 2-fold (Log2FC: -1.03) reduction in the expression of *IFF4109* in *hog1*Δ cells compared to parent, indicated on the graph. **(C)** The significantly >1.5-fold UP-regulated genes distribution in Gene Ontology (GO) slim biological processes (*p* ≤ 0.05) in *hog1*Δ cells compared to parent cells are illustrated. **(D)** The significantly >1.5-fold DOWN-regulated genes distribution in GO-slim biological processes (*p* ≤ 0.05) in *hog1*Δ cells compared to parent cells are depicted.

**FIG S2: Gating strategy and immunophenotyping of *C. auris* infected kidneys.**

**(A)** Gating strategy for fungal cell wall analysis. **(B)** Gating strategy for the flow-cytometry based myeloid cell subsets in the mouse kidney.

**FIG S3: Myeloid phagocyte accumulation of *C. auris* infected murine skin and kidneys.**

The number of the indicated myeloid phagocyte subset after epicutaneous (A), intradermal (B) and systemic (C) infection, at 72 h post-infection, with the indicated strains of *C. auris*. Data represent the mean of 4-8 individual mice. Each dot represents an individual mouse. Error bar represents mean ± SD; *****p* < 0.0001 by unpaired *t* test.
